# The effect of cellphone position on driving and gaze behaviour

**DOI:** 10.1038/s41598-021-87120-2

**Published:** 2021-04-08

**Authors:** Philip R. K. Turnbull, Safal Khanal, Steven C. Dakin

**Affiliations:** 1 University of Auckland, 85 Park Rd., Grafton, Auckland, 1023 New Zealand; 2 University of Alabama at Birmingham, 1716 University Blvd., Birmingham, AL 35233 USA; 3 UCL Institute of Ophthalmology, University College London, 11-43 Bath Street, London, EC1V 9EL UK

**Keywords:** Human behaviour, Oculomotor system

## Abstract

Legislation frequently restricts the use of cellphones while driving. Despite this, many people continue to interact with cellphones covertly while driving, typically by concealing their device in their lap. This strategy leads to frequent diversion of the drivers’ gaze from the road ahead, potentially reducing their driving performance. To evaluate the influence of cellphone use on driving, 30 participants took part in three randomly ordered 7-min virtual reality driving simulations. In each condition, drivers were presented with either (a) no cellphone, (b) a cellphone fixed to the windscreen, or (c) a cellphone positioned at lap level. Their task was to maintain road position and observe speed limits while answering maths problems (delivered intermittently via ‘text message’) and searching for external target objects. Outcome measures included speed, lane position standard deviation (LPSD), and fixation behaviour, which were compared between trials. In trials where a cellphone was present, participants shifted fixation more frequently, drove approximately 6 km/h faster, exhibited a lower LPSD and spent more time in the correct lane on the road (compared to the no-cellphone condition; all *p* < 0.001). Cellphone position influenced eye gaze behaviour, with drivers looking at the cellphone less frequently, and the speedometer more frequently. when the cellphone was in their lap compared to when the cellphone was positioned on the windscreen. Our results are consistent with participants driving more cautiously—checking speed and lane position more frequently—when they have a cellphone in the lap. Real-world driving data would be useful to determine whether this change in driving behaviour we observed is sufficient to offset the increased risk introduced by spending less time looking at the road ahead.

## Introduction

Driving a motor vehicle is a complex task demanding precise coordination of cognitive, physical, sensory, and psychomotor skills^1^. During real-world driving, the addition of a secondary activity such as cellphone use serves to divert the driver’s attention, as completion of the secondary task competes with the primary driving task for cognitive resources. As a result, there is a reduction in the driver's ability to process the critical information to the standard required by law^1,2^, impairing driving performance^3^ and increasing the risk of a traffic accident^4^. Driver distraction contributes to 14% of crashes in Australia^5^, and 9% of fatal crashes in New Zealand^6^. In 2018 alone, distraction accounted for 12 fatal crashes, 155 serious injury crashes and 956 minor injury crashes in New Zealand^7^. More concerning is that after many years of improvements, traffic injuries and deaths have recently begun increasing across the globe (e.g., a record high of 380 deaths in 2018 in New Zealand), with many attributing this to increased cellphone use while driving.

Sources of driver distraction can be both internal and external to the vehicle. While external sources of distraction (e.g., billboards) are beyond the driver’s control, in-vehicle or internal sources are frequently self-imposed and avoidable. Cellphone use is seen as a singular threat to road safety, with almost two-thirds of the drivers in high-income countries admitting to using their cellphones at some time while driving^8–10^. The wide range of engaging and complex activities available on cellphones present the potential to be a multifaceted distraction, including auditory, physical, cognitive, and emotive components. It has been reported that cellphone use leads to a much greater reduction in driving performance that other distractions, such as passenger conversations^11–14^, with the result that drivers have four times higher risk of a road collision while using a cellphone^15^.

While the use of cellphones in “hands-free” mode is anecdotally thought to be safer than the conventional mode of use, studies suggest that this is not the case^16,17^. The use of cellphones, even in hands-free mode, takes cognitive capacity away from driving, and reduces visual awareness of the surrounding environment, resulting in poorer vehicle control^18^. However, as drivers are no longer holding the phone, hands-free cellphone users are less cognizant of the impairment, but still face the same cognitive challenges, so are less likely to show compensatory behaviour (e.g., reducing their speed, or increasing their following distance^19,20^) than drivers using cellphones conventionally. Further, hands-free *texting* while driving has an even greater negative impact on driving than conducting a conversation using a cellphone because texting requires users gaze to also look at the phone^21^. In general, receiving and sending texts while driving leads drivers to spend longer and more frequent periods (as much as 4× as long) looking away from the road^22–24^. This frequent and prolonged divergence of gaze leads to up to 23-fold increase in the risk of a serious incident while driving^25^. Visual distraction while driving interfers with perceptual and motor processes and poses competiting needs for vision^26^. Consequently, drivers engaged in texting while driving are also less likely to notice external hazards, exhibit longer reaction times, and have less stable lateral and longitudinal control of their vehicles^11,14,27^.

Apart from the cognitive distraction involved in reading or composing a text message, drivers foveating a cellphone screen are reliant on their peripheral vision for vehicle control and monitoring of external hazards. Visual acuity declines rapidly with eccentricity^28,29^, and driving performance exhibits an exponential decline in reaction time and lateral vehicle control when drivers use only peripheral vision^22^.

While visual and cognitive distractions associated with cellphone divert attention away from the primary task of driving (inattention or “mind off road”^30^), engaging in competing (secondary) mental tasks while driving may increase cognitive load, depleting cognitive resources available for driving, and affecting the driving performance^26^. A real-world driving experiment found that secondary mental tasks while driving under a speed limit impair the speed control ability of the drivers and lead to increased speeds, supporting the “attentional speed control theory” that the cognitive load from the secondary tasks caused diversion of attentional resources required to control speed^31^. However, the relationship between cognitive load and driving performance remains controversial, with some studies reporting negative effects while others reporting improved driving performance^26,32^. A cognitive control hypothesis has also been suggested to imply that the effect of cognitive load on driving performance is strongly selective and task dependent, primarily affecting performance of nonpractised tasks but not of automatized tasks^26,32^.

In an attempt to minimise the increased risk of harm from operating a cellphone while driving, many countries, including New Zealand, Australia, and most states in America, have introduced varying levels of restriction of cellphone use while driving. These measures vary from restricting to hands-free use, banning interaction with a cellphone, through to banning interaction with any electronic devices with wireless communication capabilities^23,33^. However, neither legislation nor knowledge of risk^19,23^ appears to be impacting driver behaviour, with 92% of American respondents agreeing that texting while driving was dangerous, but 74% of drivers admitting to using their phone whilst on the road^33^. In Australia, one in six drivers admit to regularly text messaging while driving^24^. Rather than curtailing dangerous driving behaviour, such restrictions seem to have led to drivers taking extra efforts to avoid being caught. Approximately 80% of drivers now report using their mobile phones covertly (i.e. holding their phones out of sight to persons external to the vehicle) to avoid detection by law enforcement^23^. Because viewing a phone that is being held in this manner necessarily increases the eccentricity at which the driver is now viewing the road, the laws that sought to improve road safety may paradoxically be promoting more dangerous driving.

This study aimed to investigate whether the use of cellphone in a ‘covert’ position affects driving performance or eye gaze behaviour compared to use in a hands-free (positioned on the windscreen), or no cellphone use. We evaluated driving performance in an immersive virtual reality (VR) environment^34,35^, avoiding the risks and ethical issues associated with real-world experimentation, and affording greater control over the driving environment. We also recorded eye gaze within the virtual reality headset to compare gaze behaviours in different conditions.

## Methods

### Study participants

Thirty participants (15 male, 15 female) aged between 20 and 28 years old (mean: 23, SD: 1.7 years) were took part in the study. To be included in the study, participants needed to meet the New Zealand vision standard for driving (6/12 or better), with or without correction, and to have held a minimum of a New Zealand Class 1 (Car) Full drivers' licence for at least one year. Subjects with a history of amblyopia, strabismus, presbyopia, or a self-reported susceptibility of motion sickness were excluded from the study. Participants were fully informed about the risks and benefits of the study, gave informed consent in writing, and were free to withdraw without giving a reason at any stage. Our procedures adhered to the tenets of the Declaration of Helsinki. The study was approved by the University of Auckland Human Participants Ethics Committee (reference 021195). Participants were compensated for their time with gift vouchers.

### Study design

The study used a randomised cross-over design, with all participants completing three trials in a randomised order—each lasting 7 min—within a single visit. In each trial, the cellphone was positioned within the virtual environment mounted either on the front windshield or down by the participant’s knee (i.e. in a concealed/lap position), or there was no cellphone present. Before each test, each participant engaged in 2 min of familiarisation with the task/environment before data recording commenced. This phase served to acclimatise the participant to the virtual environment and allowed the vehicle to get to speed so that the initial period of vehicle acceleration from standstill did not confound the measures. Between conditions, participants removed the virtual reality headset and rested if necessary (mean total visit duration was 37 min, range 27–53 min). When participants were ready to begin a trial, they placed the headset on their head and aligned it with their eyes themselves, before the headset was secured in place with velcro straps.

### Simulator design

A virtual 20 km looping road, incorporating gentle left and right turns made over shallow dips and hills, was created in 3D modelling software (Blender, Stichting Blender Foundation, Amsterdam, Netherlands), and then imported into a 3D game engine (Unity 2018, Unity Technologies, San Franciso, USA). We designed the road to resemble a typical rural two-lane New Zealand road, with a large flat grass verge on either side and double-yellow centre lines, which indicate vehicles are not permitted to cross the centre line. There were no intersections or other traffic on the road. A simple 3D car model (Cicada, Retro Valorem, http://retrovalorem.wixsite.com) was modified in Blender to make it right-hand drive and incorporate additional headroom. We also added a rotating steering wheel, realistic engine sounds, and an operational dashboard with speedometer and tachometer. The car had an automatic transmission and was controlled using a steering wheel with force feedback and pedals (G920, Logitech, Lausanne, Switzerland). The force feedback added mild turn resistance, a centring force, and tactile feedback when one or more wheels were off the road.

**Figure 1:**
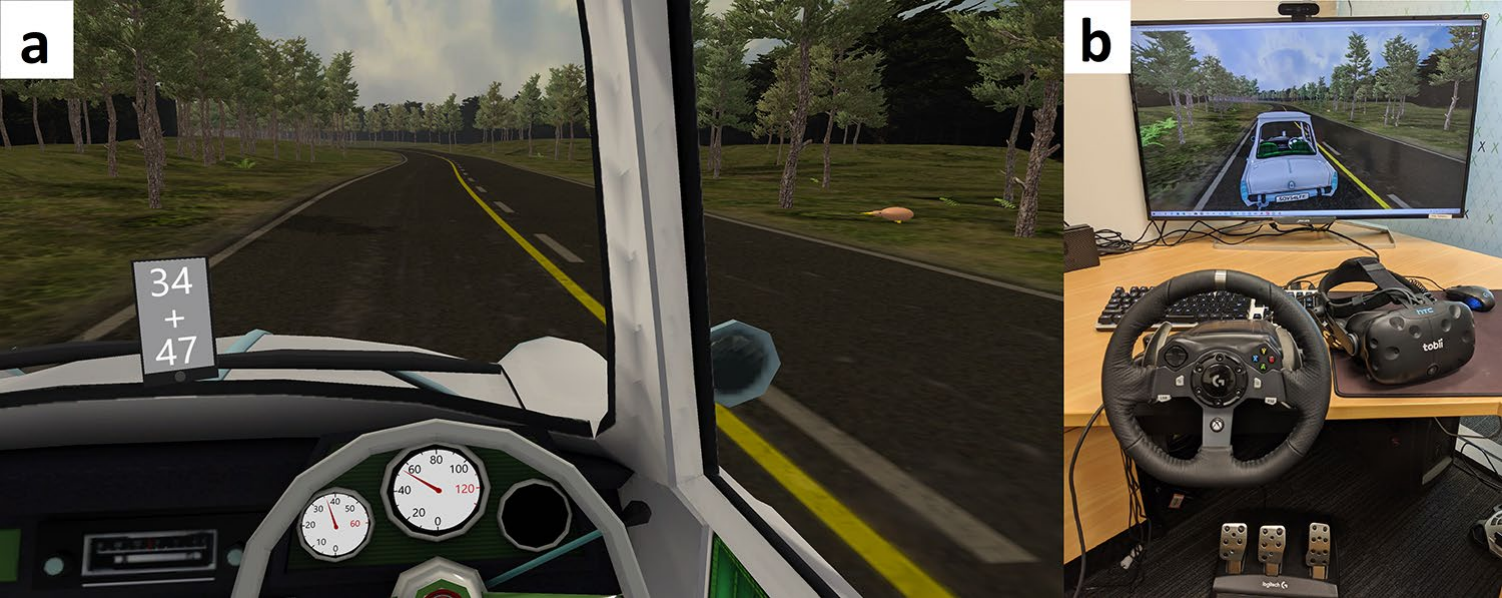
(**a**) The view from the driver's seat of the car, showing the cellphone in the windscreen position, which was displaying an addition problem that participants were tasked with solving. Also visible through the driver’s window is a kiwi, which participants counted (as an external awareness task). (**b**) The virtual reality simulator setup showing the steering wheel, pedals, and VR headset. The position of the virtual car seat, and the real-world position of the chair, steering wheel and pedals could be adjusted so that real-world proprioception felt congruous with the visual positioning of the virtual wheel. The monitor displayed real-time driving performance and gaze information to the investigators (not visible to the participant).

The driving simulator was used within a virtual reality environment, using a head-mounted display incorporating eyetracking (HTC Vive, New Taipei City, Taiwan, with Tobii eyetracking, Danderyd, Sweden, Fig. 1). Once the VR headset was aligned, stable, and fitted comfortably, the built-in infrared eyetracker was calibrated using the default five point calibration at the beginning of each trial. To maximise immersion, the participant then reached for the steering wheel and adjusted the virtual “seat position” to align the position of the real steering wheel with the model of the steering wheel in VR. The height of the virtual seat could also be adjusted so that the view over the virtual dashboard was familiar. The participant then depressed the accelerator to begin the warm-up period.

Once the trial was underway, ten “text messages” consisting of simple two-digit addition math problems were displayed over the course of the trial. These messages appeared either on the phone screen or were delivered verbally for the no-cellphone condition. An audible cue emanating from the cellphone position coincided with the delivery of each text message. Participants had to verbalise the answer, which was recorded as correct or incorrect by the investigators, who could see the maths problem (and the correct answer) on a separate display. The timing of message arrivals was randomly distributed over the 7-min trial period, with at least 30 s between each message and the end of the trial. To encourage attentive driving, participants were also tasked with spotting roadside items (custom made 3D kiwi models), which were placed randomly on the roadside around the track. The program kept track of how many kiwi were driven past, and the percentage seen was recorded per trial. There was no other traffic nor any hazards on the road, and participants were advised of the nature of the tasks ahead of time. Participants were also advised to comply with the speed limit of 100 km per hour and to follow normal road rules.

### Outcome measures

Outcomes were compared within-subject between each of the three trials with a different cellphone position. There were three categories of outcome measures for each trial. The first class of outcome measures was *car position and speed* (sampled at 90 Hz), which was used to calculate the standard deviation of lane position (SDLP). Contrary to the use of mean lane position—which would effectively force participants to drive in the centre of the lane—SDLP instead captures changes in natural lane positioning^36^. Similarly, the standard deviation of speed (SDS) was also calculated, as was the time spent speeding (i.e. faster than 100 km/h), the mean speed during the entire trial, and the duration of excursions from the correct lane, (measured from when the tyres of the vehicle touched either the left or right bounding road markers).

The second class of outcome measure was *eye gaze behaviour*, which was also captured at 90 Hz. Eye gaze was nominally classified into objects of focus: either the front windscreen, driver or passenger front side window, the dashboard, the cellphone, or elsewhere inside the car. As some changes in gaze position (e.g. from the windscreen to the cellphone) also captured intermediary targets passed over during saccade (e.g. car interior), gaze data was filtered with a rolling mode with a width of 100 ms (9 samples), based on the minimum dwell time between consecutive saccades^37^. Number of glances and total gaze duration were compared between trials, as was the mean latency time to look at the cellphone (when present) after receiving a text message.

**Figure 2:**
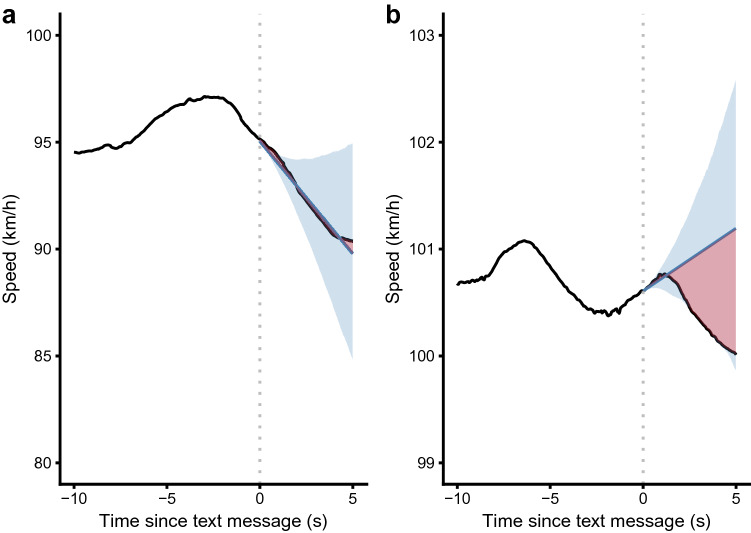
Two separate trials showing the Prophet modelling (blue line, with shaded blue area representing 95% confidence intervals) of vehicle speed. In plot (**a**), despite a large overall change in speed, there is little change in the speed trend, so the model fits well giving a mean absolute error (MAE, shown as red area) of 0.16 km/h. In plot (**b**), the participant changed their behaviour by lifting their foot off the accelerator pedal shortly after the text message arrived, which meant the model fit more poorly, yielding a higher MAE value of 1.18 km/h.

As the cellphone distraction task was intermittent and sparse (with messages appearing approximately every 40 s) compared to the data sampling rate (90 Hz), we investigated the immediate effect of receiving a text message on driving performance by comparing driving characteristics immediately prior to and following the arrival of each text message as an interrupted time-series. Measurements of speed and lane position in the 10 s leading up to a text message were fit with an additive time-series model (Fig. 2; *Prophet*, available from https://facebook.github.io/prophet/)^38^. The cross-validation function in Prophet was used to ensure an optimal fit to the data before the text message arrived. The time series function was then extrapolated a further 5 s beyond the arrival of the text message, with the mean absolute error (MAE) of the model against the observed driving behaviour recorded. The MAE of all ten text messages received during a single trial was recorded and taken as a mean MAE per trial. If driving behaviour was unaffected by the arrival of the text message, the time series would be expected to fit the post-text data well, giving a lower MAE (Fig. 2a). However, if there was a change in driving behaviour, this would be reflected as a higher MAE, as the model was unaware of the interruption to the time series (Fig. 2b). While this means that the MAE number is not useful in itself, because our analysis is within-subject and comparing between cellphone positions, the differences in the MAE should directly reflect the relative change in driving behaviour for each participant, following the arrival of the text messages.

Our final class of outcome measure was *participant task performance*, which included the accuracy of solving the maths problems and the percentage of kiwi correctly spotted.

### Statistical analysis

We used R^39^ and RStudio^40^ for all analyses. As the main outcome measures were not normally distributed, performance among trials within subject were compared using an aligned ranks transformation ANOVA, with cellphone position and participant as factors. Pairwise post-hoc comparison using Holm multiplicity correction was performed as required. The MAE of the time-series Prophet models were compared using the within-subject Friedman test, with cellphone position as a factor. The secondary outcome measures, the percentage of maths problems correct and percentage of kiwi seen, were compared using the Friedman test with all-pairs comparison testing for different factor levels according to Dwass, Steel, Critchlow and Fligner^41^. Significance was defined as *p* < 0.05.

## Results

**Figure 3:**
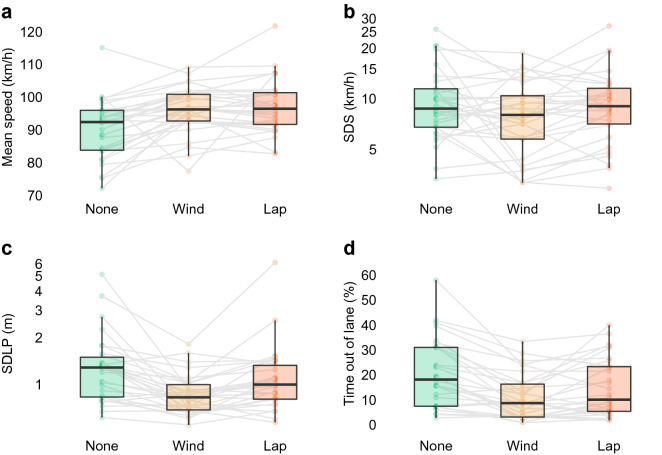
Comparison of driving behaviour when a cellphone was present (in either the Wind(ow) or Lap position) or absent. Outcome measures include (**a**) mean speed, (**b**) standard deviation of speed (SDS), (**c**) standard deviation of lane position (SDLP), and (**d**) proportion of time during the trial where any part of the vehicle was on or outside the correct lane markings. Trials where the cellphone was present were associated with faster driving, a lower SDLP, and less time spent out of lane position. Compared with having the cellphone on the windscreen, having the cellphone in the lap resulted in a higher variation in lane position and an increased amount of time with the vehicle out of the correct lane on the road.

All 30 participants completed the full 7-min trial under all three test conditions. There was a significant difference in the mean speed among the trials (F_(2,58)_ = 17.68, *p* < 0.001, Fig. 3a), with a higher mean speed in trials when cellphone was present, compared to when cellphone was absent (None—Wind: − 18.00 mean rank, t = − 5.122, *p* < 0.001, None—Lap: − 18.20 mean rank, t = − 5.179, *p* < 0.001). There was no difference in mean speed when the cellphone was in the lap compared to when it was on the dashboard (Wind—Lap: − 0.20 km/h, t = − 0.057, *p* = 0.955). While the mean speed was higher in trials with a cellphone present, there was no difference in the standard deviation of speed (SDS) among the three trials (F_(2,58)_ = 1.51, *p* = 0.230, Fig. 3b).

In terms of lane positioning, there were differences in the variation in car lane positioning across trials (SDLP, F_(2,58)_ = 17.67, *p* < 0.001, Fig. 3c). Both cellphone conditions had lower variation in lane position than the no-cellphone condition (None—Wind: 22.67 m, t = 5.893, *p* < 0.001, None—Lap: 8.73 m, t = 2.271, *p* = 0.027). The SDLP was higher when the cellphone was in the lap compared to when it was on the dashboard (Wind—Lap: − 13.93 m, t = − 3.622, *p* = 0.001). This translated into differences in the time spent out of the correct lane (F_(2,58)_ = 17.02, *p* < 0.001, Fig. 3d), as the car spent more time in the correct lane during both cellphone conditions compared with the no-cellphone condition (None—Wind: 19.33%, *t* = 5.804, *p* < 0.001, None—Lap: 11.37%, t = 3.412, *p* = 0.002). Between the two trials in which the cellphone was present, the car spent more time out of the correct lane when the cellphone was in the lap compared with when it was on the dashboard (Wind—Lap: − 7.97%, t = − 2.392, *p* = 0.020).

**Figure 4:**
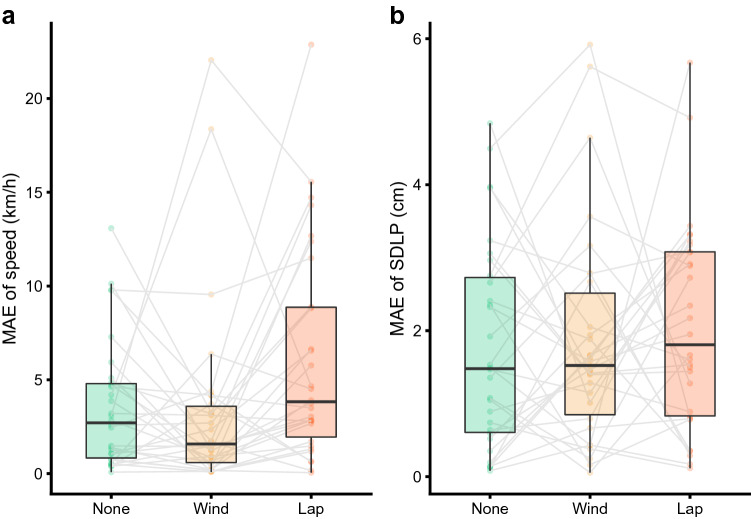
Comparison of the change in driving behaviour (mean absolute error, MAE) from the 10 s preceding—compared to the 5 s following—the arrival of a text message in terms of (**a**) vehicle speed, and (**b**) standard deviation of lane position (SDLP). We observed greater variation in speed after receiving the text message on a laptop cellphone, compared to the window position. There was no difference in SDLP between conditions.

To investigate any abrupt change in driving behaviour immediately after receiving a text message, a model was fit to driving behaviour prior to the text message arrival, enabling a comparison of the goodness of fit (mean absolute error, MAE) after receiving the text message among the three different conditions. In terms of vehicle speed, there was a significant difference among the three conditions (Chi-sq_(2)_ = 10.40, *p* = 0.006, Fig. 4a), with a greater change in speed when the cellphone was in the lap position (6.08 km/h) compared to the windscreen position (3.35 km/h, *p* = 0.032). In terms of lane positioning, there was also a difference in the change in SDLP between the three conditions after receiving the text message (Chi-sq_(2)_ = 0.47, *p* = 0.792, Fig. 4b).

**Figure 5:**
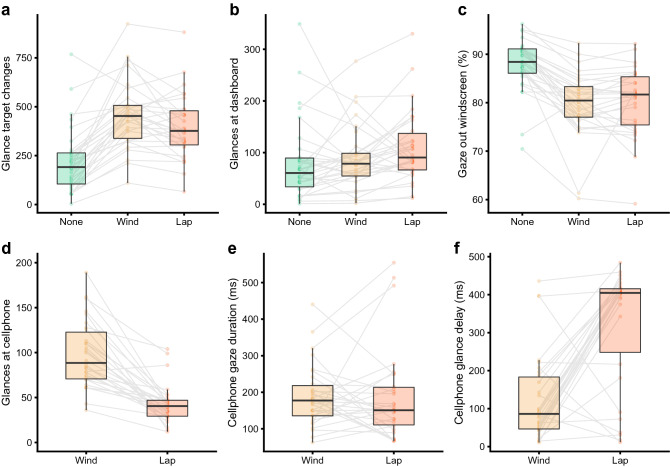
Eyegaze behaviour compared across trials, in terms of the number of glances (**a**) in total, (**b**) at the dashboard, (**c**) forwards out the windscreen, and (**d**) at the cellphone. Subfigure (**e**) shows the mean duration of each gaze at the cellphone, and (**f**) shows the time to first glance at the cellphone after the text message was delivered. The presence of a cellphone changed gaze behaviour, with less time spent looking out the window. Compared to the cellphone on the lap, participants glanced more frequently at the cellphone on the windscreen but did so for a similar total duration. However, when the cellphone was at lap height, participants delayed their glance at the cellphone after receiving the incoming text message compared to when the phone was on the windscreen.

Fixation behaviour differed across trials (F_(2,58)_ = 47.82, *p* < 0.001, Fig. 5), with fewer gaze target changes when no cellphone was present (192, IQR: 105–264) compared to when the cellphone was on either the windscreen (452, IQR: 338–507, None—Wind: − 33.63, t = − 9.396, *p* < 0.001) or in the lap position (376, IQR: 305–480, None—Lap: -25.22, t = − 7.045, *p* < 0.001). There was also a higher number of gaze changes when the cellphone was on the window compared to the lap position (Wind—Lap: 8.42, t = 2.351, *p* = 0.022). There were also differences in the number of glances at the dashboard, where the speedometer was located, (F_(2,58)_ = 7.52, *p* = 0.001), with a higher number of glances when the cellphone was in the lap (91 IQR 67–137 glances) than when the cellphone was either on the windscreen (79, IQR: 55–99, Wind—Lap: − 9.87, t = − 2.339, *p* = 0.046) or absent (61, IQR: 34–90, None—Lap: − 16.23, t = − 3.849, *p* < 0.001). There was no difference in the number of glances at the dashboard between the no cellphone and windscreen trials (None—Wind: − 6.37, t = − 1.510, *p* = 0.137). In the two cellphone trials, all but one of the participants looked at the cellphone more frequently when it was positioned on the windscreen (89, IQR: 71–123 glances) than on the lap (41, IQR: 29–47 glances, V = 464.00, *p* < 0.001). With a cellphone present, participants spent less time looking out the windscreen (F_(2,58)_ = 55.43, *p* < 0.001), and spent over twice as much time looking at the cellphone when it was on the windscreen (3.8, IQR: 2.6–6.3%) compared to when it was on the lap (1.6, IQR: 1.0–2.2%, V = 464.00, *p* < 0.001). Despite differences in the total number of glances at, and the total time spent looking at the cellphone, there was no difference in the mean cellphone glance duration (Wind: 177, IQR: 136–218 ms, Lap: 151, IQR: 111–214, V = 253.00, *p* = 0.685), nor in the amount of time spent looking forwards out the windscreen between the two cellphone positions (Wind: 80.4, IQR: 77.0–83.3%, Lap: 81.7, IQR: 77.0–83.3%, V = 204.00, *p* = 0.570). We also compared latency between the arrival of a text message and the time taken to look at the cellphone (averaged over the ten trials, per participant) in each position. There was a much greater latency when the cellphone was in the lap (405, IQR: 248–416 ms) compared to when it was on the windscreen (86, IQR: 47–183 ms, V = 38.00, *p* < 0.001).

In terms of the secondary diverted attention tasks, all participants were well engaged in all three conditions. There was no difference between trials in the proportion of kiwis seen (Chi-sq_(2)_ = 2.55, *p* = 0.279), with almost all kiwis seen in each trial condition (None: 91%, Lap: 90%, Wind: 86%). However, there was a difference in the percentage of maths problems correctly answered (Chi-sq_(2)_ = 13.85, *p* < 0.001), with an average of one additional error made when a cellphone was not present (mean 14% error rate) compared to when it was on the windscreen (6% error rate, *p* = 0.039), or in the lap position (5% error rate, *p* = 0.015).

## Discussion

Cellphones can be addictive, promoting a perceived need in their users to “remain connected”, and satisfying this urge can lead to significant distraction of drivers^42^. While many countries have introduced legislation to restrict the use of cellphones, individuals can successfully conceal phone-use by holding the phone below the window line^43^. Our hypothesis was that a phone placed in this position would impair driving performance to a greater extent than when the cellphone was positioned on the windscreen, due to the larger diversion in gaze.

Our results support this hypothesis, with several driving metrics becoming worse when the cellphone was in the lap compared to the windscreen position. First, there was a higher variation in lane position when the cellphone was in the lap, which led to a greater proportion of the time spent out of the correct lane. Vehicle positioning and heading is somewhat intuitive, but requires gaze to be forward to detect the optic flow from the external environment^44^. While some eye movement helps improve the perception of heading, the greater gaze angle required to fixate on the phone in the lap would have reduced the overall peripheral optic flow stimulus, and could explain the poorer performance when the cellphone was in the lap position^45^. There was also a significantly higher change in speed after the text message was delivered to the phone in the lap position, showing a reduction in attention being spent on speed control. Human speed perception, especially in simulators^46^, is much less instinctive that direction, and more prone to adaptation^47^, requiring frequent recalibration by glancing at a speedometer. We did observe a higher number of glances at the dashboard when the cellphone was in the lap, perhaps reflecting the difficulty in maintaining speed estimates with less available cognitive resource, causing the increased reliance on the speedometer.

These changes in driving behaviour are consistent with the cognitive load framework detailed by Engström^26^. The greater eccentricity of the phone in the lap position likely placed a higher visual load on the driver, in addition to the cognitive load introduced by solving the maths problem, diverting more attention from from the primary driving task. During both trials with the cellphone present, poorer speed control was evident, as participants drove faster and spent a greater portion of the time above the speed limit (23% of the total time with no cellphone, to almost 40% when a cellphone was present, in either position). This agrees well with the attentional speed control theory that indulging in a secondary attention-requiring task while driving compromises resources that are required to keep speed under control, resulting in an increase in driving speed^31^.

The presence of a cellphone, and its position, also influenced gaze behaviour. When on the windscreen, it received more than twice the number of glances than when on the lap. A cellphone on the windscreen presents a constant temptation to glance at it, which would be especially problematic for drivers with poorer impulse control^48^. The very brief latency to glance (median 86.2 ms) at the cellphone after receiving the text message when in the windscreen position may result from increased impulsivity when the cellphone is always in view^49^. However, this increase in the number of glances may not affect driving safety. A real-world driving study has shown that although drivers spent approximately 4% of their time looking at the in-vehicle information systems, they spent on average only half a second per glance, and they do so without reducing glances to the mirrors, driving equipment, or the centre of the road^50^. The comparative additional latency to glance at the cellphone when in the lap position could reflect an awareness of the additional cognitive distraction the position poses. Participants may be aware that looking further away from the road to check the phone would be disruptive to driving performance, so they deliberately waited for a suitable time to check the phone (e.g. immediately after negotiating a corner).

The high accuracy achieved in the maths tasks shows that participants were adequately attending to the secondary diverted attention tasks, and the higher score in the cellphone-present trials likely reflects the advantage of a lesser cognitive load and consequently an ability to devote greater resources for visual load, as the participants had the added benefit of visualising numbers on the cellphone and not needing to memorise the addends. Interestingly, despite having less time available to view their surroundings when looking at a cellphone, there was no difference in the number of kiwi seen between the trials. This task, in addition to being a way to ensure participant engagement, served as a proxy ‘hazard identification’ scenario, so it is reassuring that essentially all targets were correctly identified during all trials. Modification of gaze behaviour helps to explain this observation, as we found greater latency in looking at the phone after receiving the text message when it was in the lap, compared to when it was on the windscreen. This finding suggests that participants spent time waiting for an opportune moment and ensuring the car was positioned correctly, before taking their eyes off the road. This behaviour would include looking forward to potential hazards/targets.

A significant limitation of this study is our use of a virtual reality simulator, and a simulated text message as a distraction. A comparison of simulator versus real-world performance showed that while distractions may have a larger influence on driving performance in a simulator, comparative simulator performance, like our study design, is reasonably consistent with real-world driving^51^. Our study also has limited ability to extrapolate to real-world safety, as there was no real threat to our participants, and our environment was static; if there was no hazard present before gazing away from the road, one was not going to appear suddenly. This is perhaps the most significant issue with distracted attention while driving. While known risks can be theoretically adjusted for through modified behaviour, unseen or unpredictable hazards can arise when the gaze is averted from the road. Additionally, our diverted attention task (answering text messages consisting of maths problems) would potentially present a diminished sense of urgency in checking the cellphone—answering a maths problem is unlikely to be as exciting as participating in a group chat, for example. Also, while the maths problem served as an information processing cognitive task, this too is likely different from what a cellphone using distracted driver may typically experience, and gaze becomes increasingly forward-looking with increasingly complex distraction tasks^52^. However, we found good engagement and comparative performance in the diverted attention tasks across trials. Nonetheless, it is unlikely that our results will directly extrapolate into real-life driving, and the results are best viewed as within-subject comparisons.

Most vehicle crashes involve younger drivers^53^, who are known to routinely underestimate road hazards^54^, and admit to frequently sending text messages while driving^55^. As the number of young drivers on the road increases over time, the number of vehicle accidents attributable to distraction from cellphone-use is likely to continue to rise. The legislation we have in place is intended to prevent dangerous driving behaviour, but the addictive nature of cellphone use is leading drivers—despite being aware of the legislation and risks—frequently engaging in behaviour while driving that reduces their capacity to operate a vehicle responsibly. Furthermore, investigations into the impact of legislation designed to deter cellphone use while driving are clearly warranted.

## Conclusion

It is established that cellphone use while driving distracts the driver from the primary task of operating the vehicle, and puts them and others at increased risk of harm. However, legislation to prevent or restrict cellphone use runs counter to the additive nature of these devices, especially in younger age groups. We found that, while viewing a cellphone in a concealed position, drivers demonstrated inferior driving behaviour, with poorer control over lane position and speed. Further research, ideally incorporating more realistic external hazards, would be useful to determine whether this legislative changes create a safer driving environment for all.
